# Community-led data collection using Open Data Kit for surveillance of animal African trypanosomiasis in Shimba hills, Kenya

**DOI:** 10.1186/s13104-019-4198-z

**Published:** 2019-03-18

**Authors:** Sarah A. O. Wamwenje, Ibrahim I. Wangwe, Nicodemus Masila, Caroline K. Mirieri, Lillian Wambua, Benard W. Kulohoma

**Affiliations:** 1 Centre for Biotechnology and Bioinformatics, University of Nairobi, Nairobi, Kenya; 2 International Centre of Insect Physiology and Ecology, Nairobi, Kenya; 3 Kenya Tsetse and Trypanosomiasis Eradication Council (KENTTEC), Kwale County, Kenya; 4 Directorate of Veterinary Services, Vector Regulatory and Zoological Services, Makindu, Kenya; 5 School of Biological Sciences, University of Nairobi, Nairobi, Kenya

**Keywords:** Community-led, Surveillance, Smartphone, Trypanosomiasis

## Abstract

**Objective:**

In Sub-Saharan Africa, there is an increase in trypanosome non-susceptibility to multiple trypanocides, but limited information on judicious trypanocide use is accessible to smallholder farmers and agricultural stakeholders in disease endemic regions, resulting in widespread multi-drug resistance. Huge economic expenses and the laborious nature of extensive field studies have hindered collection of the requisite large-scale prospective datasets required to inform disease management. We examined the efficacy of community-led data collection strategies using smartphones by smallholder farmers to acquire robust datasets from the trypanosomiasis endemic Shimba hills region in Kenya. We used Open Data Kit, an open-source smartphone application development software, to create a data collection App.

**Results:**

Our study provides proof of concept for the viability of using smartphone Apps to remotely collect reliable large-scale information from smallholder farmers and veterinary health care givers in resource poor settings. We show that these datasets can be reliably collated remotely, analysed, and the findings can inform policies that improve farming practices and economic wellbeing while restricting widespread multi-drug resistance. Moreover, this strategy can be used to monitor and manage other infectious diseases in other rural, resource poor settings.

**Electronic supplementary material:**

The online version of this article (10.1186/s13104-019-4198-z) contains supplementary material, which is available to authorized users.

## Introduction

Animal African trypanosomiasis (AAT), a life-threatening neglected tropical disease that affects cattle, goats and sheep, caused by trypanosomes parasites (*Trypanosoma congolense*, *Trypanosoma brucei*, and *Trypanosoma vivax*) is endemic to Shimba hills, in Kwale County, Kenya, a resource poor setting inhabited by smallholder farmers [1]. Tsetse flies, the vectors that transmit trypanosomes, are widely spread across in 38 of the 47 counties in Kenya, occupying approximately 138,000 km^2^ (23% of the country), putting livestock and people at risk of infection [1]. Chemotherapy is the main strategy for disease management, but trypanosomes are progressively becoming non-susceptible to multiple drugs [2–4]. The increase in parasite non-susceptibility is largely driven by inappropriate and prolonged use of trypanocides [1, 5]. Thus, providing information on judicious trypanocide use to smallholder farmers and agricultural stakeholders in this disease endemic region could significantly reduce the development and spread of multi-drug non-susceptibility [6]. However, this is hindered by inadequate data on: prevalence of multi-drug non-susceptible trypanosomes, perceptions of smallholder farmers about disease management and use of trypanocides, and access to veterinary services in resource poor endemic settings [7]. The huge economic expenses and laborious efforts associated with extensive field studies prohibit collection of the requisite large-scale prospective data. We aimed to evaluate the efficacy of community-led data collection using smartphones for robust information collation to monitor cattle trypanocide non-susceptibility, and also to answer pertinent trypanosomiasis management associated research questions using data from smallholder farmers in disease endemic region of Shimba hills, Kwale County, Kenya.

## Main text

Conventional data collection tools requiring physical presence of investigators are expensive, and significantly slowdown data collection, analysis and results dissemination [8, 9]. Mobile phone applications (Apps) for remote data collection show great promise for their use for data collection in rural settings [10, 11], but remain largely untested for disease surveillance in marginalised endemic areas [12]. Apps are faster, inexpensive, and have quality control mechanisms ensuring consistency in data quality [13–15]. Basic feature cell phones, as opposed to a smartphones, can also be used for financial transactions, engage users/participants, assess outcomes, pinpoint geographic locations, and measure levels of social contact and connectedness [16]. The International Telecommunication Union estimates nearly 100% global mobile phone subscription rates by over 5 billion users; with over 70% residing in developing countries [17, 18]. This provides new avenues to robustly monitor and evaluate health outcomes; understand the social-economic, behavioural and environmental factors influencing health and illness; and provide forecasts that allow early intervention to avert disease epidemics [1, 16, 19, 20].

Kenya has 100% mobile phone penetration, and a 67% smartphone penetration rate, which is 4 times higher than Africa’s average (18%) [21, 22]. Internet connectivity via smartphones accounts for 83% of web traffic, well above the global average of 52% [23].

Although performing financial transactions using mobile phones is predominant [22], there is increasing use in agricultural sector to improve profitability and farming practices among Kenyan smallholder farmers. For example, M-Farm, provides real-time national market prices of agricultural produce thereby allowing farmers to get the best prices and eliminating unscrupulous middlemen; and iCow that provides livestock husbandry advice to smallholder communities in rural remote areas without access to veterinary and extension officers [24, 25].

Community engagement when implementing mobile phone-based strategies is important for gaining trust, educating on use and importance of data collected, and easy reporting of feedback [26]. Kwale County is one of the poorest regions, with a low literacy level (57%) in Kenya [27]. We examine the efficacy of community-led data collection using smartphones for monitoring cattle trypanocide non-susceptibility and to provide data on smallholder farmer perceptions on trypanosomiasis in Shimba hills, Kwale County, Kenya. We also examine whether this strategy of data collection was robust enough to collect information on inclusivity, since data on gender inequality are harder to collect and constitute the “silences” in smallholder farming in resource poor settings.

### Methods

#### Study site and data collection

This pilot study was part of a larger study evaluating evolutionary markers of trypanocide non-susceptibility conducted among two smallholder livestock farming communities of different ethnicities from Mbegani and Kizibe in Shimba hills, Kwale County, Kenya [1]. We obtained informed written consent and interviewed veterinary health care givers and smallholder farmers to gain insight and contextualise factors related to accessibility, timeliness and relevance of mobile health (m-Health) Apps in dissemination of agricultural information. We collected information on trypanocide use for disease management, drug non-susceptibility by their cattle after treatment, and knowledge on trypanosomiasis management (Additional file 1). We anonymised all personal data using unique identification numbers. Data was securely stored on an access-restricted database at the University of Nairobi’s Centre for Biotechnology and Bioinformatics.

#### Data application

We used Open Data Kit (ODK) an open-source smartphone App development software to create a data collection App [28]. ODK has a user-friendly web interface for designing the mobile App forms and programming simple logic [29]. During data collection filled forms were initially stored on the phone’s memory, prior to storage at the remotely located centralised database for further analysis. The App ensured the automatic storage of the mobiles’ geographical locations, and pictures and videos taken during data collection. The collected data was not altered prior to transmission. The App data entries were converted to Excel spreadsheets for further statistical analysis [30, 31].

#### Multiple correspondence analysis

We used multiple correspondence analysis (MCA), a data reduction technique which permits the identification of complex patterns in a dataset of categorical variables [32], to determine variation between the farmers in both villages, and generate a graphic representation of our findings using the response data collected. We included only information that gave insight on farmers perceptions to trypanosomiasis and trypanocide use to be able to understand the effect these perspectives have on farming productivity. This analysis was implemented using SPSS statistics for Windows (version 24.0), NY: IBM Corp.

### Results

**Fig. 1:**
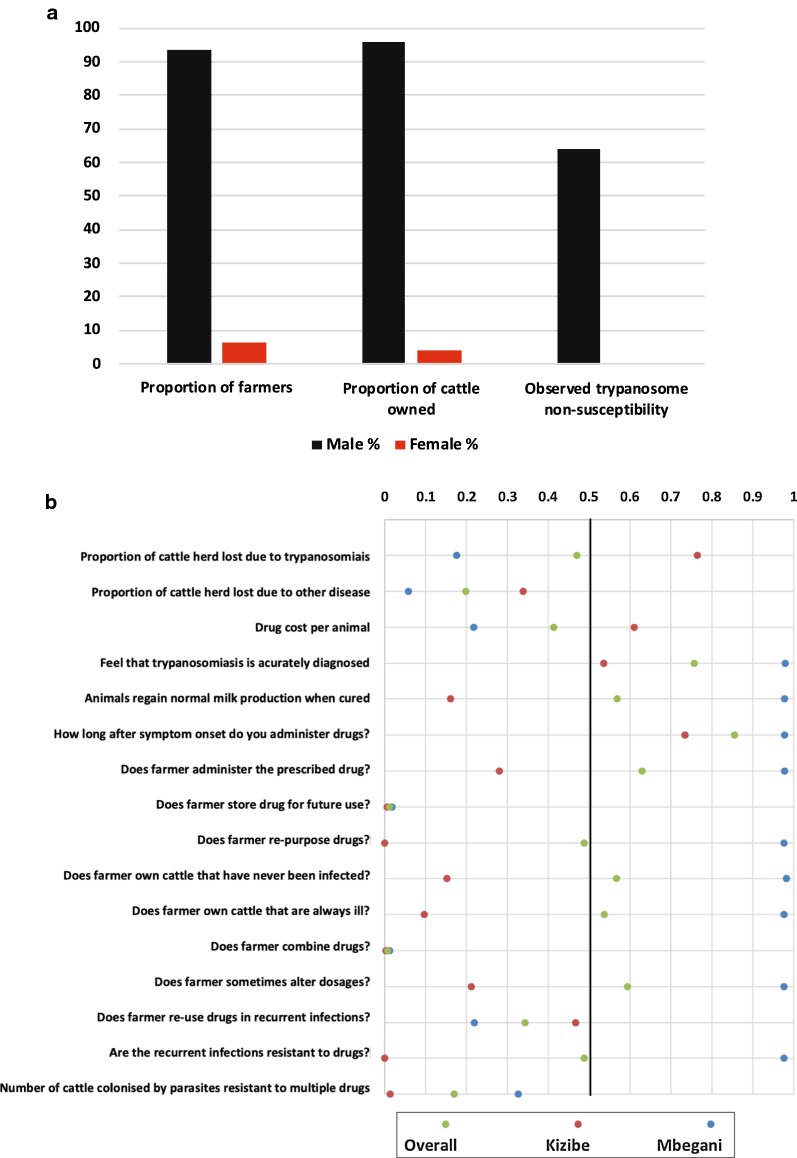
**a** Gender inclusivity in cattle husbandry in Kwale. The proportion of female farmers was smaller (6%, n = 3 of 50), and they also had a lower proportion of the overall livestock ownership (4%, n = 8 of 211). **b** Farmers perceptions on livestock trypanosomiasis. Shows the perception of farmers based on results from multiple correspondence analysis (MCA) conducted separately for Mbegani and Kizibe. The x-axis scale reflects the degree to which farmers disagreed (0) to mostly agreed (1) on the research questions asked during data collection. Responses from Kizibe (red) are distinguished from those from Mbegani (blue), and also the overall mean values (green) is also determined

We conducted a pilot study on the efficacy of a community-led data collection approach by conveniently sampling from two communities of smallholder farmers (n = 47) from: Kizibe (n = 23) and Mbegani (n = 24) to acquire information on trypanocide non-susceptibility and perceptions of smallholder farmers on trypanosomiasis. There was a disproportionately lower number of female smallholder farmers (6%, n = 3), who also had less cattle ownership (4%, n = 8) compared to their male counterparts (Fig. 1a). These female farmers were all from Mbegani, and their cattle were not infected with non-susceptible trypanosomes. This is in contrast to male farmers who owned cattle (64%) infected with non-susceptible trypanosomes. Our results from multiple correspondence analysis show variations in perception of trypanosomiasis management, and use of trypanocides between the two communities of smallholder farmers (Fig. 1b). Smallholder farmers in Mbegani lost fewer cattle due to trypanosomiasis, spent less on trypanocides, regained milk production earlier after treating livestock, keenly followed drug prescription instructions, and noticed that recurrent infections are resistant to treatment.

A large proportion of smallholder farmers and veterinary health care givers interviewed (94%, n = 47) welcomed the smartphone App developed on the ODK platform as a method of trypanosomiasis data collection. The farmers with dissenting views about using the App were illiterate. Our multiple correspondence analysis results show variation between the two communities from the overall mean, which would not be detected if knowledge on community ethnicity was not considered.

### Discussion

Kwale is one of the most resource poor Counties in Kenya. Kizibe and Mbegani, in Shimba hills, Kwale, are trypanosomiasis endemic regions with varying economic capacities and different farming practices, which have made understanding temporal trends in trypanocide non-susceptibility challenging. Previous studies have used generalised cross-sectional survey approaches that lumped together findings from different communities in Kwale making it challenging to pinpoint subtle differences in farmers perceptions and disease management practices [33]. We established that it is feasible to implement robust community-led data collection using a smartphone application by smallholder farmers in Kwale to improve knowledge and management of trypanosomiasis and trypanocide non-susceptibility. Our results from the initial tranche of the community-led data collection using the ODK-based mobile App was able to highlight a disparity between gender inclusivity and cattle ownership among smallholder livestock farmers in Kwale, suggesting that women are less economically empowered compared to men. This finding supports previous studies showing that in sub-Saharan Africa, female farmers have many setbacks that hinder their agricultural productivity, which is 23–66% lower than that of male counterparts. Improving access to productive resources would increase global agricultural yields by up to 30%, raise economically viable activities by up to 4%, and reduce global hunger for up to 150 million people [34, 35]. Although female smallholder farmers in Shimba hills have smaller cattle herds compared to their male counterparts, trypanocide non-susceptibility is absent in their herds suggesting that they exercise better farming practices, since their cattle were non-susceptible to multiple trypanocide drugs. The farmers in Kwale commonly rear the Boran cattle [36]. Thus, it is unlikely that this difference is due to a few smallholder farmers’ livestock becoming trypanotolerant. This is a remarkable observation considering all farmers practice free-range farming in common community grazing and watered fields, and hence the animals come into close contact, and have similar rates of exposure to disease vectors.

We used multiple correspondence analysis (MCA) to highlight subtle variation in farming practices by the smallholder farmers in Kizibe and Mbegani, which could be used to inform trypanosomiasis management. Similar approaches have been used to assess animal African trypanosomiasis (AAT) vulnerability in cattle-owning communities of sub-Saharan Africa [37]. Our results emphasise the potential to evaluate pertinent research questions that were previously intractable at vast scales, and unprecedented due to economic restrictions and the laborious nature of field data collection exercises. ODK-based Apps can be customised to local dialects, have user-friendly web-interfaces, are cost-effective, and receive good technical support making them favourable for use in resource poor settings. The ease of use will ensure acceptance by local communities as the only dissenting voices were from the illiterate farmers. We established that smallholder farmers with dissenting views (4%, n = 2) were illiterate, and had reservations on embracing information technology. However, these farmers and other stakeholder, for example veterinary healthcare workers and extension officers can be taught how to use the App during prospective community engagement activities or have reliable literate proxies assist them with data entry.

Mobile phone affordability and technology uptake has increased tremendously in Kenya [22], suggesting the importance of information technology tools to smallholder farmers in resource poor settings for access to critical farming information. Our study provides proof of concept for the viability of using mobile Apps to remotely collect reliable large-scale information from smallholder farmers and veterinary health care givers in resource poor settings. This information can be collated remotely, and analysis based on the evidence available can be used to inform policies that improve farming practices and economic wellbeing while restricting widespread multi-drug resistance. Moreover, the community-led data collection using Open Data Kit for surveillance of animal African trypanosomiasis approach, can be used to monitor and manage other infectious diseases in numerous settings, and shows a great potential of improving socio-economic status of smallholder farmers in rural areas. We conclude that technological advances provide new avenues for remotely collecting data from disease endemic areas. These methods are easy to adopt in developing countries, and their success, sustainability and scalability relies on community engagement during implementation.

## Limitations

The limitation in this pilot study was focus on a restricted set of questions to establish proof of concept, but other pertinent research questions based on study design can be included for prospective research. We also anticipate some recall bias during data collection. We currently underpowered to estimate the extent of this bias, but anticipate that a much larger dataset from prospective data collection will reduce confounding during analysis. This study was conducted using datasets from smallholder farmers due to insufficient data from the small number of veterinary healthcare workers. Another setback was the intermittent mobile network coverage by some mobile service providers. We were able to circumvent this challenge by transmitting information to the central database once connected to a strong network.

## Additional file

**Additional file 1.** Questionnaire used to develop the ODK-based mobile App. The App questions were divided into two: (i) questions to animal health care workers (ii) questions to smallholder farmers.

## Abbreviations

AAT: animal African trypanosomiasis
App: mobile phone application
m-Health: mobile health
ODK: Open Data Kit
MCA: multiple correspondence analysis

## References

[1] Wangwe II, Wamwenje SA, Mirieri C, Masila NM, Wambua L, Kulohoma BW (2018). Modelling appropriate use of trypanocides to restrict wide-spread multi-drug resistance during chemotherapy of animal African trypanosomiasis. Parasitology.

[2] Genois MM, Paquet ER, Laffitte MC, Maity R, Rodrigue A, Ouellette M, Masson JY (2014). DNA repair pathways in trypanosomatids: from DNA repair to drug resistance. Microbiol Mol Biol Rev MMBR.

[3] Unciti-Broceta JD, Arias JL, Maceira J, Soriano M, Ortiz-Gonzalez M, Hernandez-Quero J, Munoz-Torres M, de Koning HP, Magez S, Garcia-Salcedo JA (2015). Specific cell targeting therapy bypasses drug resistance mechanisms in African trypanosomiasis. PLoS Pathog.

[4] Simarro PP, Cecchi G, Paone M, Franco JR, Diarra A, Ruiz JA, Fevre EM, Courtin F, Mattioli RC, Jannin JG (2010). The Atlas of human African trypanosomiasis: a contribution to global mapping of neglected tropical diseases. Int J Health Geogr.

[5] Chitanga S, Marcotty T, Namangala B, Van den Bossche P, Van Den Abbeele J, Delespaux V (2011). High prevalence of drug resistance in animal trypanosomes without a history of drug exposure. PLoS Negl Trop Dis.

[6] Liebenehm S, Bett B, Verdugo C, Said M (2015). Optimal drug control under risk of drug resistance—the case of African animal trypanosomosis. J Agric Econ.

[7] Holt HR, Selby R, Mumba C, Napier GB, Guitian J (2016). Assessment of animal African trypanosomiasis (AAT) vulnerability in cattle-owning communities of sub-Saharan Africa. Parasit Vectors.

[8] de la Vega R, Miro J (2014). mHealth: a strategic field without a solid scientific soul. A systematic review of pain-related apps. PLoS ONE.

[9] DeCamp M (2015). Ethical issues when using social media for health outside professional relationships. Int Rev Psychiatry.

[10] Heinonen R, Luoto R, Lindfors P, Nygard CH (2012). Usability and feasibility of mobile phone diaries in an experimental physical exercise study. Telemed J E Health.

[11] van Heerden AC, Norris SA, Richter LM (2010). Using mobile phones for adolescent research in low and middle income countries: preliminary findings from the birth to twenty cohort, South Africa. J Adolesc Health.

[12] King C, Hall J, Banda M, Beard J, Bird J, Kazembe P, Fottrell E (2014). Electronic data capture in a rural African setting: evaluating experiences with different systems in Malawi. Glob Health Action.

[13] Ganesan M, Suma Prashanta S, Jhunjhunwala A (2012). A review on challenges in implementing mobile phone based data collection in developing countries. J Health Inform Dev Ctries.

[14] Bell AR, Ward PS, Killilea ME, Tamal ME (2016). Real-time social data collection in rural bangladesh via a ‘microtasks for micropayments’ platform on android smartphones. PLoS ONE.

[15] Kipf A, Brunette W, Kellerstrass J, Podolsky M, Rosa J, Sundt M, Wilson D, Borriello G, Brewer E, Thomas E (2016). A proposed integrated data collection, analysis and sharing platform for impact evaluation. Dev Eng.

[16] Cnossen R, Heetderks W, Kumar S, Nilsen W, Pettigrew R, Patrick K, Riley B, Tippett P, Topol E, Volpp K. White paper: data collection and mobile technologies. Precision medicine initiative: building a large US research cohort. 2015. https://www.nih.gov/sites/default/files/research-training/initiatives/pmi/data-collection-mobile-technologies.pdf. Accessed 8th Jan 2019.

[17] WHO. Handbook on the use of Mobile Phone data for Official Statistics. UN Global Working Group on Big Data for Official Statistics. 2017. https://unstats.un.org/bigdata/taskteams/mobilephone/HandbookonMobilePhoneDataforofficialstatistics-DraftNov2017.pdf. Accessed 8th Jan 2019.

[18] WHO, eHealth GOf (2011). mHealth: new horizons for health through mobile technologies. Second global survey on eHealth.

[19] Wahl B, Cossy-Gantner A, Germann S, Schwalbe NR (2018). Artificial intelligence (AI) and global health: how can AI contribute to health in resource-poor settings?. BMJ Glob Health.

[20] Aanensen DM, Huntley DM, Feil EJ, Al-Own F, Spratt BG (2009). EpiCollect: linking smartphones to web applications for epidemiology, ecology and community data collection. PLoS ONE.

[21] TECHZiM. Kenya has 67% smartphone penetration, way above the continent’s figures. 2014. https://www.techzim.co.zw/2014/04/kenya-67-smartphone-penetration-way-continents-figures/. Accessed 6th Mar 2019.

[22] Communication Authority of Kenya. Mobile money transfers Hit Ksh. 2 Trillion As Penetration Shoots To 100 Per Cent. 2018. https://ca.go.ke/mobile-money-transfers-hit-ksh-2-trillion-as-penetration-shoots-to-100-per-cent/. Accessed 8th Jan 2019.

[23] JUMIA Kenya. Smartphones: the gateway to a better life. KENYA Mobile White Paper 2018. 2018. https://jumia.co/nl-templates-kenya/uploads/mobile-report/Jumia_MW18_White_Paper.pdf. Accessed 5th Mar 2019.

[24] Mbiti I, Weil DN (2013). The home economics of e-money: velocity, cash management, and discount rates of M-Pesa users. Am Econ Rev.

[25] Qiang CZ, Kuek SC, Dymond A, Esselaar S. Mobile applications for agriculture and rural development. The World Bank Report Number 96226. 2012;1:1–536. Accessed 10th Jan 2017.

[26] Stanton MC, Mkwanda SZ, Debrah AY, Batsa L, Biritwum NK, Hoerauf A, Cliffe M, Best A, Molineux A, Kelly-Hope LA (2015). Developing a community-led SMS reporting tool for the rapid assessment of lymphatic filariasis morbidity burden: case studies from Malawi and Ghana. BMC Infect Dis.

[27] Government KC. Education and literacy in Kwale. 2019. http://www.kwalecountygov.com/index.php?option=com_content&view=featured&Itemid=936. Accessed 22nd Jan 2019.

[28] Raja A, Tridane A, Gaffar A, Lindquist T, Pribadi K (2014). Android and ODK based data collection framework to aid in epidemiological analysis. Online J Public Health Inform.

[29] Steiner A, Hella J, Gruninger S, Mhalu G, Mhimbira F, Cercamondi CI, Doulla B, Maire N, Fenner L (2016). Managing research and surveillance projects in real-time with a novel open-source eManagement tool designed for under-resourced countries. J Am Med Inform Assoc JAMIA.

[30] Singh H (2013). Mobile data collection using an android device. IJCST.

[31] Flannery M, Budden DM, Mendes A (2015). FlexDM: simple, parallel and fault-tolerant data mining using WEKA. Source Code Biol Med.

[32] Abdi H, Valentin D, Salkind N (2007). Multiple correspondence analysis. Encyclopedia of measurement and statistics.

[33] Ohaga SO, Kokwaro ED, Ndiege IO, Hassanali A, Saini RK (2007). Livestock farmers’ perception and epidemiology of bovine trypanosomosis in Kwale District, Kenya. Prev Vet Med.

[34] Walker E. Male farmers are up to 3 times more productive than female farmers. ONE. 2015. https://www.one.org/international/blog/male-farmers-are-up-to-3-times-more-productive-than-female-farmers/. Accessed 10th Jan 2019.

[35] Bandama M. The crucial role of women in African agriculture. Farmer’s weekly. 2016. https://www.farmersweekly.co.za/opinion/by-invitation/the-crucial-role-of-women-in-african-agriculture/. Accessed 10th Jan 2019.

[36] Maichomo MW, Kosura WO, Gathuma JM, Gitau GK, Ndung’u JM, Nyamwaro SO (2009). Economic assessment of the performance of trypanotolerant cattle breeds in a pastoral production system in Kenya. J S Afr Vet Assoc.

[37] Holt HR, Selby R, Mumba C, Napier GB, Guitian J (2016). Assessment of animal African trypanosomiasis (AAT) vulnerability in cattle-owning communities of sub-Saharan Africa. Parasit Vectors.

