# Supplementary material for: Community-led data collection using Open Data Kit for surveillance of animal African trypanosomiasis in Shimba hills, Kenya
Source: BMC Res Notes. 2019 Mar 18;12:151. doi: 10.1186/s13104-019-4198-z (PMC6423862; doi:10.1186/s13104-019-4198-z)
Supplement: Supplementary file 1 — Additional file 1. Questionnaire used to develop the ODK-based mobile App. The App questions were divided into two: (i) questions to animal health care workers (ii) questions to smallholder farmers. [file 13104_2019_4198_MOESM1_ESM.pdf]

Questionnaire used to evaluate knowledge on trypanosomiasis and trypanocide use in Shimba Hills, Kwale County, Kenya.

Note: the score of 1 – 10 is used to measure the extent to which the respondent gives weight to the response on this scale.

<u>Section to be filled by veterinary officer</u>

Name of area (locale) where attached –
Which villages in your locale are worst hit by the trypanosomiasis?

....

Which areas report the least infection rates?

....

What is the average cost of a single drug regimen per animal?

....

Is this affordable to the farmers?
A. Yes
B. No

Score: (1 = least likely ; 10 = most likely)

1	2	3	4	5	6	7	8	9	10

Disease and drug awareness data

1. How often are you contacted by the farmers over trypanosomiasis?
   A. Daily
   B. Once a week
   C. Twice a week
   D. Once a month
   E. Other (*Please specify*)..

2. At what point do the farmers contact you?
   A. Immediately they notice illness
   B. After self-medicating
   C. As a last resort
   D. Other (*please specify*)..

3. Are farmers generally open and ready to provide information?
   A. yes
   B. No

Score: (1 = least likely ; 10 = most likely)

1	2	3	4	5	6	7	8	9	10

4. If NO, why?
   A. Myths and suspicions
   B. Ignorance
   C. By choice with no particular reason
   D. Other (*please specify*)...

Diagnosis and prescription patterns

5. How many animals in a week do you think you treat with nagana?
   A. 1
   B. 2
   C. 3
   D. Other (*please specify*)...

6. How do you go about diagnosing an animal?
   A. I know it is nagana from experience
   B. I collect samples for lab analysis and confirmation
   C. I rely on my colleagues
   D. I rely on the farmers knowledge

7. Are you always confident in your final diagnosis*?*
   A. YES
   B. NO

Score: (1 = least likely ; 10 = most likely)

1	2	3	4	5	6	7	8	9	10

8. Do you experience any difficulties during diagnosis? *If YES, please give details.*
   A. YES
   B. NO

Score: (1 = least likely ; 10 = most likely)

1	2	3	4	5	6	7	8	9	10

9. If an APP was developed to aid your diagnosis process, would you use it?
   A. YES
   B. NO

Score: (1 = least likely ; 10 = most likely)

1	2	3	4	5	6	7	8	9	10

10. Which drug(s) are you most likely to prescribe?

A. Homidium
B. Isometamidium
C. Diminazene
D. Other (*please specify*)……………………………………………………………………………………

11. At what stage of the disease do you prescribe the named drug (s)?
    A. Before onset (prophylaxis)
    B. Early onset
    C. Late disease stage
    D. Other (*please specify*)……………………………………………………………………………………

12. What is your reason for choosing the above-named drug(s)?
    A. Most available
    B. Most affordable
    C. Most effective
    D. Other (*please specify*)……………………………………………………………………………………

Drug use and efficacy and toxicity

13. Do the farmers stick to the regimen given?
    A. Yes
    B. No

Score: (1 = least likely ; 10 = most likely)

1	2	3	4	5	6	7	8	9	10

14. Have you noticed any seasonal variation in drug sensitivity?
    A. Yes
    B. No

Score: (1 = least likely ; 10 = most likely)

1	2	3	4	5	6	7	8	9	10

15. Have you ever prescribed any of the drugs used to treat nagana for another condition other that nagana in cattle?
    A. Yes
    B. No

Score: (1 = least likely ; 10 = most likely)

1	2	3	4	5	6	7	8	9	10

16. Do you know of any local remedies used by local farmers as an alternative to chemical therapies?
    A. Yes
    B. No

Score: (1 = least likely ; 10 = most likely)

1	2	3	4	5	6	7	8	9	10

17. What do you think has caused the multidrug resistance?
    A. over-prescription
    B. self-prescription by farmers
    C. farmers are not completing regimens
    D. Other (*please specify*)..

18. What are some of the toxic effects evidently caused by the drugs?
    A. photosensitivity
    B. skin necrosis
    C. hair loss
    D. Other (*please specify*)..

19. Are they also seen during combinations?
    A. Yes
    B. No

Score: (1 = least likely ; 10 = most likely)

1	2	3	4	5	6	7	8	9	10

20. Any other livestock other than cattle visibly resistant to the trypanocidal drugs?
    A. Goats
    B. Sheep
    C. donkeys
    D. Other (*please specify*)..

<u>Section to be filled by farmer</u>

Name of village –

Number of cattle owned –

What is the cost of a single drug regimen per animal?

The burden

1. Is there any noticeable difference in productivity between infected and healthy animals?
    A. No difference
    B. Sick animals are obviously less productive
    C. Sick animals are more productive
    D. Both groups are equally productive

2. If there is a difference, what is the difference and to what extent

....

Score: (1 = least likely ; 10 = most likely)

1	2	3	4	5	6	7	8	9	10

....

3. Out of the animals that fall sick, how many have still died even after receiving medication (please give a number)?
   A. All
   B. None
   C. Half
   D. Other (*please specify*)..

4. What are the other causes of cattle fatalities in your herd besides nagana (please give number of fatalities)?
   A. East Coast Fever
   B. Contagious Bovine Pleuro-pneumonia (CBPP)
   C. I don't know
   D. Other (*please specify*)..

5. What are the proportion of cattle affected by disease other than Nagana, please give figures?
   A. All
   B. None
   C. Half
   D. Other (*please specify*)..

6. What are your options eventually when an animal is not responding to medication?
   A. Slaughter
   B. Sell
   C. Await death
   D. Other (*please specify*)..

7. How do you determine that your animal is infected with Nagana?
   A. I know it is nagana from experience
   B. I wait for the vet to confirm
   C. I rely on fellow farmers knowledge
   D. Other (*please specify*)...

8. Are you always confident in your final diagnosis?
   C. YES
   D. NO

Score: (1 = least likely ; 10 = most likely)

1	2	3	4	5	6	7	8	9	10

9. After recovery, do the animals regain full productivity?
   A. Yes
   B. No
   C. Other (*please specify*)..

Score: (1 = least likely ; 10 = most likely)

1	2	3	4	5	6	7	8	9	10

Drug prescription, use and toxic effects

10. How long after symptom onset do you wait before administering medication?

A. immediately
B. I wait for a few days
Other (if B, how many days)...
   A. I give immediately
   B. It takes a few days as I have to raise funds to buy drugs
   C. I pre-medicate to prevent infection
   D. Other (*please specify*)..

11. Who recommends the drugs you use?
   A. Myself
   B. Vet
   C. Fellow farmers
   D. Other (*please specify*)..

12. Which of the three drugs do you prefer using (*please confirm brand names*)?
   A. Homidium
   B. Isometamidium
   C. Diminazene
   D. No preference

13. Do you stick to the prescribed drug regimen?
   A. Yes
   B. No
   C. If no, please explain why...

Score: (1 = least likely ; 10 = most likely)

1	2	3	4	5	6	7	8	9	10

14. Do you prolong the use of the drug? *(Use for longer than prescribed).*
    A. Yes
    B. No
    C. If yes, please explain why...

Score: (1 = least likely ; 10 = most likely)

1	2	3	4	5	6	7	8	9	10

15. When the animal's health is restored, do you store the medication for use later?
    A. Yes
    B. No

Score: (1 = least likely ; 10 = most likely)

1	2	3	4	5	6	7	8	9	10

16. Have you ever received different dosage levels for the drugs from different vets?
    A. Yes
    B. No

Score: (1 = least likely ; 10 = most likely)

1	2	3	4	5	6	7	8	9	10

17. Have you ever used the drugs as a combination?
    A. Yes
    B. No
    C. If yes, please give details..

Score: (1 = least likely ; 10 = most likely)

1	2	3	4	5	6	7	8	9	10

18. How many times in a year does the infection recur thus necessitating the need to buy the drug(s)?
    A. Once
    B. Twice
    C. Thrice
    D. Other (*please give details*)..

19. What are the toxic /negative effects of the drug on the cattle?
    A. photosensitivity

B. skin necrosis
C. hair loss
D. Other (*please specify*)..

20. Of your drugs of choice (and combinations) in which doses do you commonly prefer to administer?
    A. As prescribed by vet
    B. As prescribed by pharmacist
    C. As I have always done
    D. As advised by fellow farmers

21. Over time, has the dosing changed?
    A. Yes
    B. No

Score: (1 = least likely ; 10 = most likely)

1	2	3	4	5	6	7	8	9	10

22. Are you using the medication to treat anything else other than nagana?
    A. Yes
    B. No
    C. If yes (*please give details*)..

Groups of livestock

23. Do you have any cattle in your herd that have never needed medication (***non-colonised***)?
    A. Yes
    B. No

Score: (1 = least likely ; 10 = most likely)

1	2	3	4	5	6	7	8	9	10

24. (***colonised by susceptible strains***) Are there any cattle in your herd recurrently infected and a particular drug (or particular drugs) seems to be working?
    A. Yes
    B. No
    C. If yes, give details on number of cattle, drug (s), order of drugs, (any combination) and dosage..

25. Do the doses change when in combination if administered separately?
    A. Yes
    B. No

Score: (1 = least likely ; 10 = most likely)

1	2	3	4	5	6	7	8	9	10

26. Do you have any cattle in your herd that are recurrently infected and all particular drugs seem to be working?
    A. Yes
    B. No
    C. If yes (*please give details*)..

Score: (1 = least likely ; 10 = most likely)

1	2	3	4	5	6	7	8	9	10

27. How are they administered?
    A. Combination
    B. Separately
    E. Other (*please specify*)..

28. (***colonised by resistant strains***) Do you have cattle that are not responding to all the medication?
    A. Yes
    B. No

Score: (1 = least likely ; 10 = most likely)

1	2	3	4	5	6	7	8	9	10

29. (***colonised by strains resistant to two drugs***) Do you have cattle that have fallen ill then recovered after you used two drugs (which failed) and then you administered the third and it worked?
    A. Yes
    B. No
    C. If yes (*please give details*)..

Score: (1 = least likely ; 10 = most likely)

1	2	3	4	5	6	7	8	9	10

30. Do you use drug combinations often?
    A. Yes
    B. No
    C. If yes (*please give details*)..

Score: (1 = least likely ; 10 = most likely)

1	2	3	4	5	6	7	8	9	10

31. What is the average cost of a single drug regimen per animal?

....

32. Do you find the cost of the prescribed trypanocides affordable to you?
   A. Yes
   B. No

Score: (1 = least likely ; 10 = most likely)

1	2	3	4	5	6	7	8	9	10
